# Relationship between muscle power, muscle volume and limb length in healthy male and female adolescents

**DOI:** 10.3389/fspor.2024.1457948

**Published:** 2025-01-07

**Authors:** Souhail Bchini, Nadhir Hammami, Dhouha Moussaoui, Taoufik Selmi, Najla Mhimdi, Anissa Bouassida, Abderraouf Ben Abderrahman, Ismail Laher, Juan Del Coso, Hassane Zouhal

**Affiliations:** ^1^ Research Unit (UR22JS01) “Sport Sciences, Health and Movement”, High Institute of Sport and Physical Education of Kef, University of Jendouba, Kef, Tunisia; ^2^ Higher Institute of Sport and Physical Education of Ksar Said, University of la Manouba, Manouba, Tunisia; ^3^ Department of Anesthesiology, Pharmacology and Therapeutics, The Universityof British Columbia, Vancouver, BC, Canada; ^4^ Sport Sciences Research Centre, Rey Juan Carlos University, Fuenlabrada, Spain; ^5^ Movement, Sport, Health and Sciences Laboratory (M2S), UFR APS, University of Rennes 2-ENS Cachan, Rennes, France; ^6^ Institut International des Sciences du Sport (2I2S), Irodouer, France

**Keywords:** muscle strength, muscle volume, gender, teenager, jumping ability

## Abstract

**Objective:**

Muscle power is essential for the activities of daily living. Muscle power production depends on numerous factors such as muscle size and length, muscle architecture and fiber type and varies with age during growth. The association between muscle power output during a jump and lower limb muscle volume and length in adolescents is largely unknown. This study determined the relationships between muscle power developed during a countermovement jump with lower limb muscle volume and length in adolescents aged between 16 and 19 years.

**Methods:**

Forty healthy adolescent males (*n* = 20) and females (*n* = 20) aged 16 to 19 years underwent a counter-movement jump (CMJ) test. Muscle power (MP) during the jump was calculated using the Gomez-Bruton equation. Lower limb muscle volume (MV) and length were calculated in both sexes using anthropometric methods. Pearson correlation was used to assess the associations between variables. Independent-sample *t*-tests were used to compare anthropometric and muscle performance data between males and females. Cohen's *d* was used to determine the size of the differences.

**Results:**

There were differences in all anthropometric variables between males and females (*p* < 0.001). CMJ height (*d* = 4.45; *p* = 0.001) and MP (*d* = 4.74; *p* = 0.001) were greater in males than in females (*p* < 0.001). These differences persisted when jump performance was normalized to the MV (*d* = 1.05; *p* = 0.01) and length (*d* = 4.07; *p* = 0.001). There were correlations between MV and MP for males and females, with a significant correlation between limb length and MP for males (*r* = 0.55; *p* = 0.002).

**Conclusions:**

This study indicates that MV and length are associated with MP production during a CMJ in adolescents aged between 16 and 19 years, suggesting that these factors may be important determining factors for vertical jumping performance during adolescence. The sex-differences in jump performance variables persisted after normalization by MV and length, suggesting that MV and length did not entirely explain the sex difference in muscle power output during a CMJ.

## 1 Introduction

Physical performance is strongly related with age and sex during childhood and adolescence (1). Anthropometric characteristics and motor skills varies with sex and maturation (2), and sex likely a key factor in determining athletic achievements (3). The interaction between environmental and biological factors may be an important determinant of sex-based differences in physical ability evident at early ages (4). Muscle power output during intense exercise activities is considered a key component of musculoskeletal performance and is the basis for physical performance and the development of motor skills in children and adolescents (5). Muscle power, defined as the product of force and velocity, drives high-intensity, short-duration anaerobic tasks, such as maximal running speed and jumping (6). Muscle power can be used in measuring and tracking physical performance over time to determine maturation changes or the effectiveness of training programs (7). The Wingate test, different types of jumps performed on force plates, isokinetic dynamometry and a myriad of other field tests have been traditionally used to evaluate muscle power noninvasively in exercise (8). Among them, lower limb muscle power estimated from the height during a maximum vertical jump, is considered one of the easiest and more valid forms of assessing muscle power (9). The most commonly employed vertical jump test in sports sciences is the counter-movement jump (10). The vertical velocity of the centre of mass (CoM) determines the height achieved in a vertical jump at the time of take-off. Therefore, the key mechanical variable for vertical jump performance occurs during the flexion-extension of the lower limbs that precede to the jump (11, 12). In other words, lower limb muscles create the power (the flexion-extension should be done at the highest possible velocity to apply the maximum value of force per unit of time) to generate the impulse and this is directly associated with the height obtained during the jump.

Although skeletal muscles are the main contributors for power production during a jump, other passive components of the lower limbs, such as tendons, can also contribute to the generation of power (13). Numerous studies have highlighted the influence of growth and maturation on muscle power (14) and differences in the evolution of muscle power between the sexes (15, 16). The sex difference in power performance is influenced by anthropometric and task-specific factors as well as morphological characteristics of muscles (17). Among them, muscle mass, leg volume, muscle architecture, muscle fibril length and muscle fibre type distribution are all major contributors to muscle power during intense exercise activities and collectively could explain a large portion of sex differences in muscle power (14). Interestingly, the sex differences in the morphological factors that affect muscle power during growth is the result of the effects of of hormones such as the thyroid hormone, oestrogen, and testosterone (18).

Lower limb muscle volume may be one of the most significant factors explaining the sex differences in muscle power output in adults (19) and children (20). By using longitudinal research measuring changes in short-term power output in participants from 10 to 12 years of age, De Ste Croix et al. (21) observed that thigh muscle volume positively influenced muscle power developed in the Wingate test for both males and females. However, these authors reported no significant differences between the sexes for either peak or mean power developed during the Wingate test, suggesting that sex differences in muscle power were not evident at this age. In addition, Bchini et al. (20), also reported that thigh muscle volume was positively associated with muscle power developed during different jumps (squat jump, counter-movement jump and countermovement jump with free arms) in participants aged 10–12 years, but that sex-differences in muscle volume and jump performance were already evident at this age.

The current literature indicates an important influence of muscle mass on power production during short-term all-out exercise activities during childhood, but does not address the influence of sex-differences in muscle volume and muscle power. This is important because differences in hormone regulation between sexes peaks at 16–19 years of age (22). Given the role of hormones such as testosterone on muscle mass development during puberty (16), studying adolescents between 16 and 19 years of age may be key to identifying differences in anthropometric and muscle power performance between males and females and to determine the importance of muscle volume in muscle power production in each sex. Additionally, beyond muscle volume, lower limb length may contribute to muscle power production as a longer lower limb may implicate lengthier muscle fibrils, while this variable has not been previously investigated. Our study aims to determine the relationships between muscle power developed during a countermovement jump with lower limb muscle volume and length in male and female adolescents aged between 16 and 19 years. We hypothesized that a) lower limb muscle volume and limb length would be determining factors of muscle power developed during a jump in adolescent males and females, and b) muscle power output would be similar in male and female adolescents after normalization of muscle power by muscle volume and length, suggesting that sex-differences in power performance could be the result of sex-differences in anthropometric variables.

## 2 Methods

### 2.1 Overall description of the design and procedures

We evaluated the relationships between lower limb muscle volume and leg length of male and female adolescents with muscle power during a jump using linear regression and correlation analyses. Tests were conducted in the afternoon (between 14:00 to 16:00 h) to rule out potential effects of circadian regulation. Each individual completed three attempts for the counter-movement jump (CMJ), and the maximum jump height of these three attempts was chosen for the statistical analysis. An Optojump plate with infrared beams was used to record each CMJ height calculated from flight time. Muscle power (MP) was calculated according to the equation previously described by Gomez-Bruton et al. (5) based on jump height and the participant's body mass. Lower limb muscle volume (MV) and length were calculated using anthropometric methods.

Two weeks before the experiment began, participants were familiarized with the experiment and its procedures during the first visit. On this visit, anthropometric measurements were performed and the familiarisation with the CMJ test was performed by explaining in detail the characteristics of the jump and by letting the participants execute jumps of increasing intensity until they executed a maximal jump with a proper technique. At the second visit, the CMJ was performed after a standardized warm-up (15 min of jogging and dynamic stretching supervised by expert fitness coach). Standardized verbal encouragement was given by the same experimenter by using the commands “Try to jump as high as possible” before the jump and “Well done!” and after each jump to aid maximum performance in each jump.

### 2.2 Participants

We used G*Power Version 3.1.9.6, (Dusseldorf, Germany) (23) to calculate the optimal sample size for our study, employing the independent-sample *t*-tests. Expected effect size was determined with reference to the studies by Ghimire et al. (24). An effect size of 0.91, a statistical power (1−β) of 0.80, and an alpha error rate (*α*) of 0.05 were used to calculate that the participant group should consist of a minimum of 38 participants. Following this calculation, 40 adolescents (20 males and 20 females) between the ages of 16 to 19 years were recruited for this study. The physical characteristics of the participants are presented in Table 1. All participants were healthy (in possession of a medical certificate of good health status). Eligible participants had to (a) be free of injuries at the time of the study, (b) have no history of lower limb muscle injury in the three months prior to registration, (c) have no history of lower limb joint injury in the previous six months, and (d) be free of delayed-onset muscle soreness at the test session. In addition, participants were encouraged not to engage in vigorous-intensity exercise and to a avoid any pain-relieving strategies 48 h prior to the measurement. Participants were excluded if they had a history of chronic musculoskeletal or cardiorespiratory disease, or any physical condition at the time of recruitment that may affect their ability to jump. Participants were not involved in any type of structured training. All participants included in this study regularly engaged in a physical education class for 2 h a week as scheduled in the Tunisia national curriculum guidelines. Participants (those older than 18 years of age) or their parents (for those younger than 18 years of age) were fully informed about the objectives of the study and tests procedures and signed a written informed consent. The study was carried out in accordance with the Helsinki Declaration of Human Experiments. The local research ethics committee of the High Institute of Sport and Physical Education of Kef, University of Jendouba, approved the protocol (code number b15-2022, authorized on December 25, 2023).

**Table 1:** Mean ± SD of anthropometric variables for 20 males and 20 females aged 16 to 19 years old.

	Males	Females	Δ M/F (%)	*p* value	Cohen's *d*
Age (year)	17.55 ± 1.09	17.10 ± 1.02	2.56	*p* = 0.48	0.12
Body mass (kg)	66.6 ± 11.6	60.3 ± 8.6	10.3	*p* = 0.007	1.98
Body fat (%)	18.3 ± 6.2	27.2 ± 4.5	32.6	*p* = 0.002	3.85
Body height (cm)	176 ± 5.2	163 ± 7.7	7.9	*p* = 0.001	5.19
Body mass index (kg/m^2^)	22.45 ± 3.56	22.65 ± 3.21	0.9	*p* = 0.64	0.06
Lower limb length (cm)	89.2 ± 5.4	84 ± 6.9	6.2	*p* = 0.01	2.09
Lower limb muscle volume (l)	7.4 ± 0.7	5.5 ± 0.5	35.3	*p* = 0.001	3.25

ΔM/F: differences between males and females.

## 3 Measures

### 3.1 Anthropometry

Anthropometric characteristics including body height, body mass (BM), body fat percentage (% fat) and lower limb MV and lower limb length were collected by a single experienced researcher to ensure uniformity and accuracy. Body mass (model TBF 105; Tanita Corporation of America, Inc., Arling Heights, IL, USA) and body height (Holtain stadiometer, Croswell, Crymych, Pembrokeshire, UK) were measured to the nearest 0.1 kg and 0.1 cm, respectively. Lower limb length and circumferences were measured using a standardised anthropometric kit (Harpenden, Sweden). Lower limb length was obtained from the centre of the tibial plafond to the top of the femoral head. The upper thigh circumference, middle thigh circumference, under-kneecap circumference, maximum calf circumference, intercondylar distance and ankle circumference were measured with a millimetre tape. Bicipital, tricipital, subscapular, supra-iliac skinfolds and quadriceps, and calf skinfold thicknesses were measured according to techniques recommended by the International Biological Program (25) using a skinfold calliper (Harpenden skinfold calliper, CMS Instruments, London, UK). The corporal density was assessed using the methods of Durnin & Rahaman (26) using skinfold measurements, which was used to calculate whole body fat mass (25).

For males, body density was measured in accordance with Equation 1:

(1) Bodydensity=1.1765−0.0744(log10ΣS)

For females, body density was measured in accordance with Equation 2:

(2) Bodydensity=1.1567−0.0717(log10ΣS)

Where S is the sum of the skinfolds measured, in accordance with Equation 3:

(3) Massfat=(4.95/bodydensity4.5)100

Total lower limb volume was calculated using formula developed by Jones & Pearson (27). The lower limb bone volume was calculated from the intercondylar diameter of the knee according to Shephard et al. (28) and using Equation 4:

(4) Totallimbvolume=(∑C2)⋅L/62.8

Where ∑C2 is the sum of the squares of the five circumferences of the corresponding limb the fat volume is calculated according to Equation 5:

(5) Fatvolume=(∑C/5)⋅(∑S/2n)L

Where ∑S is the sum of four skinfolds for the lower limb (front of mid-thigh, back of mid-thigh, back of calf and outside of calf), and “n” represents the number of skin folds measured.

The bone volume was calculated according to Equation 6:

(6) Bonevolume=π⋅(F⋅D)2⋅L

Where D is the femoral intercondylar diameter, F is a geometric factor (0.235 for the lower limb), and L is the limb length as measured above.

Lower limb muscle volume was ultimately calculated based on Jones & Pearson (27) formula, in accordance with Equation 7:

(7) Musclevolume=totallimbvolume–(fatvolume+bonevolume)

#### 3.1.1 Peak muscle power and vertical jump height

CMJ testing was performed during the spring school break of 2023. After 15 min of warm-up exercises, jump height was quantified using an Optojump plate (Optojump, Microgate, Bolzano, Italy), which consists of 2 parallel bars placed approximately 1 m apart and parallel to each other. The optical system transmits infrared light 1–2 mm above the floor and, when the light is interrupted by the feet, the unit triggers a timer within 1 millisecond, which allows the measurement of flight time during the jump (29). Optojump bars were connected to a portable computer, and the Optojump software (version 3.01.0001) allowed jump height quantification from flight time (t) and acceleration due to gravity (g) using the formula h = g × t^2^/8 (30) (Equation 8). The best result was kept for analysis, and there was a 45-second recovery time between trials. Participants began the test by standing upright with their hands on their hips and were then instructed to flex their knees (∼90°) as quickly as possible and then jump as high as possible. Vertical jump and body mass values were used to calculate muscle power. Peak muscle power was calculated according to Gomez-Bruton et al. (5) using Equation 8:

(8) Power(W)=(54.2)*(jumpheight[cm])+34.3*(bodymass[kg])–1520.4)

### 3.2 Statistical analyses

Descriptive statistics (mean and standard deviations) were calculated for each of the variables after normal distribution was tested and confirmed using the Shapiro–Wilk test. The sphericity was tested and confirmed by the Mauchly test. Bi-variate Pearson's correlation coefficients (r) were calculated to evaluate the correlations between muscle power, muscle volume and lower limb length among males and females. The magnitude of the correlation was classified as follows: ≤0.1, trivial; >0.1–0.3, small; >0.3–0.5, moderate; >0.5–0.7, large; >0.7–0.9, very large; and >0.9–1.0, almost perfect (31). Linear regression analysis was also calculated between the peak muscle power and lower limb length and muscle volume. Independent-sample *t*-tests were used to compare data on males and females. In addition, effect size (Cohen's *d*) analysis was used to determine the size of the differences between males and females, and interpreted using the following thresholds: <0.20 (trivial); 0.20–0.60 (small); 0.60–1.20 (moderate); 1.20–2.0 (large); 2.0–4.0 (very large); and >4.0 (extremely large) (32). The difference between males and females in each variable (*Δ*M/F) was calculated using Equation 9:

(9) ΔM/F(%)=(valueofmales–valueoffemales)/valueoffemales

The level of statistical significance was set at *p* < 0.05. All statistical analyses were performed using SPSS software (version 22; IBM, Armonk, NY, USA).

## 4 Results

The anthropometric characteristics of all male and female participants are presented in Table 1. A statistically significant difference between males and females was present for body mass, body height and in lower limb length. Adolescents males had higher MV and lower percentage of body fat than their female counterparts.

The values obtained from CMJ tests in both sexes are shown in Table 2. There were significant differences in vertical jumping performance and muscle power, with higher values in all variables for males than females. Very large effect sizes were observed for CMJ and MP. When muscle power was normalized to lower limb length, males still obtained better jump performance variables than females. After normalization to muscle volume, males still exhibited better jump performance variables than female although the effect size was reduced from very large (without normalization) to small (with normalization).

**Table 2:** Vertical jump height and muscle power in absolute values and normalized by body characteristics in males and females aged 16 to 19 years old.

Variable	Male	Female	Δ M/F (%)	*p* value	Cohen's *d*
Vertical jump height (cm)	31.5 ± 7.3	20.7 ± 4.3	51%	<0.001	4.45
MP (W)	2,579 ± 447	1,681 ± 270	53%	<0.001	4.74
MP/MV (W/L)	34.7 ± 60.4	30.7 ± 60.01	12.79%	<0.05	1.05
MP/LL (W/cm)	28.9 ± 5.11	20.1 ± 4.07	43.14%	<0.001	4.07

*Δ*M/F: differences between males and females.

MP, muscle power; MV, muscle volume of lower limbs in litre; LL, lower limb length.

**Figure 1:**
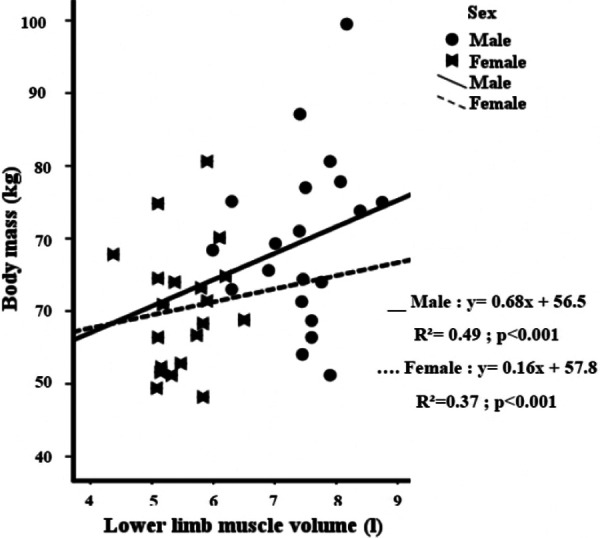
Relationship between lower limb muscle volume and body mass in adolescent males and females.

**Figure 2:**
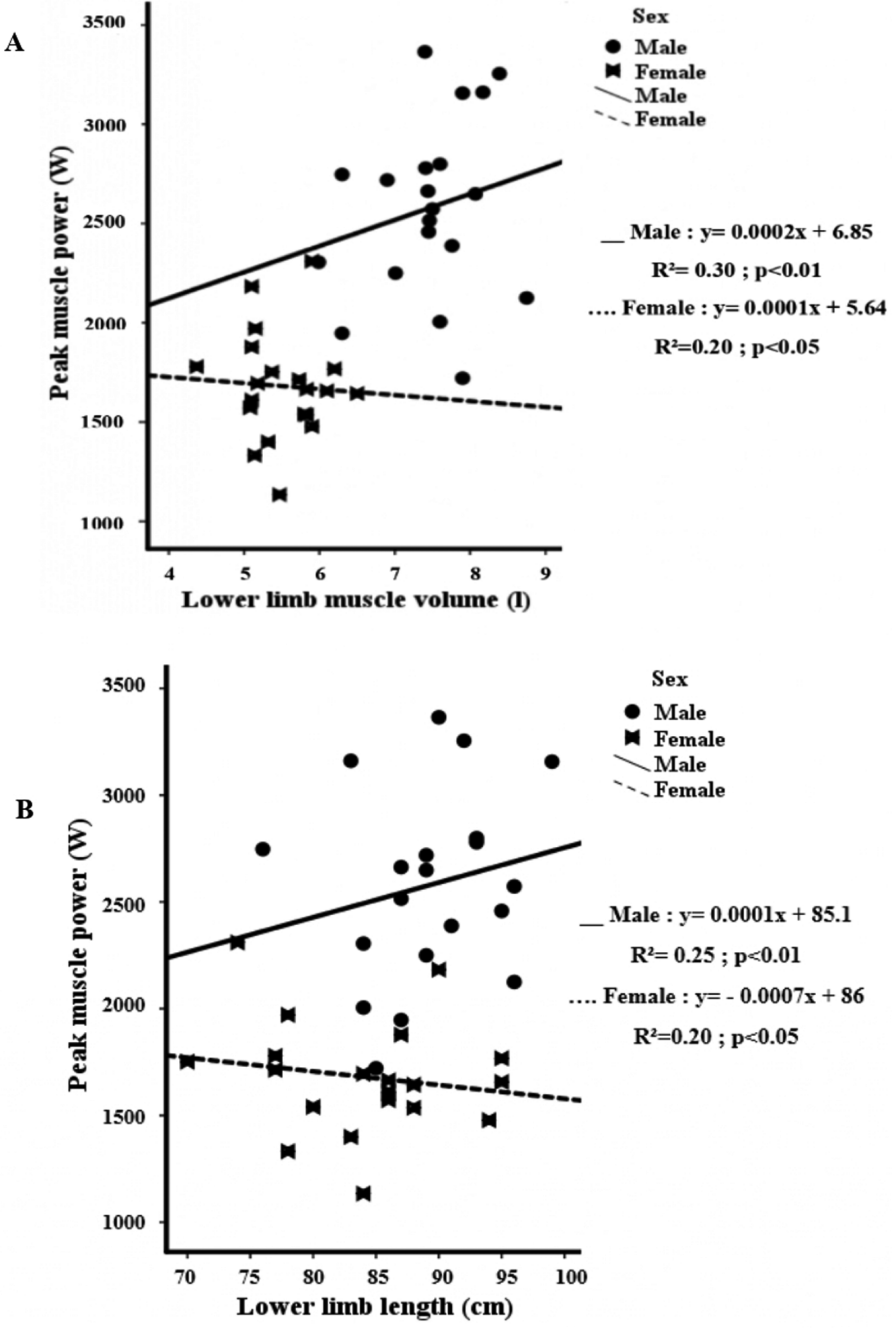
**(A)** Relationship between peak muscle power and lower limb muscle volume, **(B)** relationship between peak muscle power and lower limb length for adolescent males and females.

There were statistically significant associations between body mass and muscle volume for males (*r* = 0.70; *p* = 0.001) and females (*r* = 0.61; *p* = 0.004) as shown in Figure 1. Additionally, there were also significant associations between MV and MP for males (*r* = 0.54; *p* < 0.01) and females (*r* = 0.44; *p* < 0.05) (Figure 2A). Similarly, there were statistically significant associations between lower limb length and MP for males (*r* = 0.55; *p* = 0.002; Figure 2B).

## 5 Discussion

Our study aimed to determine the relationships of muscle power developed during a countermovement jump with lower limb muscle volume and length in male and female adolescents aged between 16 and 19 years. We postulated that muscle volume and limb length could be determining factors of muscle power in adolescent males and females, and they could explain sex dependent jump performance at this age. The present study revealed significant differences in terms of vertical jump height and muscle power between males and females. Additionally, there were strong correlations between muscle volume and lower limb length with peak muscle power, but these correlations were positive in males and negative in females. This data suggests that muscle volume and lower limb length is a determining factor for muscle power and jump performance in athletes aged 16–19 years. Nonetheless, the influence of muscle volume and lower limb length on the development of muscle power during a jump may differ between males and females. In males, the observed association between peak power and muscle volume or lower limb length aligned with our hypothesis, as larger muscles and longer limbs are typically linked to greater power. However, the association in females appeared counterintuitive, likely reflecting a complex interplay of biomechanical and physiological factors. For example, in men, longer muscles often indicate larger muscle fibers, which partly explains their greater strength compared to women (33). However, the relationship between muscle length and fiber size appears to be less pronounced or less well-defined in women (33). Additionally, larger muscles may demand more complex neuromuscular coordination for effective activation. In women, suboptimal muscle coordination might limit their ability to produce peak power output, especially when recruiting longer fibers, particularly constraining the potential of participants with longer lower limbs (34). Lastly, although we examined physically active adolescents with no formal training background, it is possible that males may participate in more non-sport, power-specific activities that females that allow them to better develop power relative to their anthropometric conditions (35). All these are hypothesis that should be tested in future studies.

In this vein, muscle volume has a greater impact on muscle performance in males, while females with moderate values of muscle volume could also exhibit high muscle performance values. These sex differences in jump performance variables persisted after normalization by lower limb muscle volume and length, suggesting that lower limb volume and length did not entirely explain the differences in muscle power output between male and female adolescents. It is probable that other factors such as muscle architecture and muscle fibre type distribution, which were not measured in this study, may also contribute to sex differences in jump performance we observed. Differences in the concentration of hormones such as the thyroid hormone, oestrogen, and testosterone between males and females during childhood and adolescence not only affects muscle mass differences but also other variables associated with muscle power production such as myosin heavy chain expression and the contractile properties of muscle (18). Our study reveals that male participants were 7.9% (i.e., 13 cm) taller and 10.3% (i.e., 6.3 kg) heavier than females participants. Additionally, compared to males, females had a higher body fat percentage (−32.6%), while males had a higher muscle volume (35.3%). Our results are similar to those reported by Wells (36), who found that hormonal differences observed at puberty led to significant increases in body fat percentage in females and increases in muscle mass in males. In addition, Kanehisa et al. (37) reported that these physiological changes were associated with puberty, including increased testosterone levels, and thus increased muscle mass in males compared to females, especially in fast twitch fibres. In the other hand, our results reveal that males had a lengthier lower limbs (6.2%) than females, which likely aided higher muscle power during jumps, as a longer limb is associated with longer muscle fibres and improved force production (38).

Our results revealed significantly greater CMJ performance and peak muscle power in males than in females. The difference in vertical jump performances between the sexes could be attributed to changes in body composition, particularly increases in the percentage of muscle fibres with the increase in leg length and leg muscle volume in boys after the age of 13 years (39). This result resembles those of earlier studies, which showed that in comparison to females, males exhibited higher eccentric and concentric strength and power as well as greater peak power during the concentric phase of CMJ (40, 41). Our study is novel because it indicates, for the first time, the large influence of muscle mass in power production during short-term all-out exercise activities during adolescence, complementing the data reported in adults (19) and children (20). These changes make adolescence as key period of growth where differences in anthropometric variables and muscle power performance are evident in males and females. Interestingly, sex differences persisted when jump performance variables were normalized to muscle volume (12.8%) and to lower limb length (43.1%), and agrees with the findings of Bchini et al. (19) in adults and in children (20).

However, the normalization of muscle power performance data by muscle volume reduced the effect size of the males-females comparison from very large to small, suggesting that a large portion of the sex-difference in performance is associated with the sex-differences in muscle mass, as we initially hypothesised. Other factors, such as muscle architecture and muscle fibre type distribution (which were not measured in our study), are other potential contributing factors for the differences in power performances in adolescent males and females (18). Moreover, our results indicate significant correlations between MV and MP for both sexes. Similarly, significant correlations were found between lower limb length and MP for males. These results are in agreement with those reported by O'Brien (42) of moderate–strong relationships between thigh muscle volume (MV) and lower limb short-term muscular power in both sexes. In line with our hypothesis, the results of our study indicate that muscle volume and limb length may be major determining factors of muscle power and vertical jumping performance in adolescents.

## 6 Study limitations

This study has several limitations that need to be addressed. First, the standard anthropometry methods used in the study may have affected the accuracy of the muscle volume measurements. Using other methods for measuring muscle volume could potentially yield different results, and using more advanced techniques could provide more improved data on muscle volume measurements. Second, the lack of measurements of maturation or hormone levels in the participants is another limitation of the study. These factors can play a significant role in muscle development and performance, and the lack of their measurements in the study may have overlooked important influences on the outcomes. Especially for participants over 18 years of age, as they are likely to have completed the majority of their maturation processes. In addition, no measurements were made of other parameters that may affect jump performance, such as muscle architecture and muscle fibre distribution, were performed. Such data could have provided additional insights into the mechanisms underlying jump performance and sex-differences, and may have shed light on the specific physiological characteristics contributing to an individual's ability to jump. Future studies should include measurements of muscle architecture and fiber type distribution to complete the understanding on how muscle mechanic factors determine muscle power differences between the sexes during childhood and adolescence. Although our sample size calculations were designed to minimize the likelihood of Type I and Type II errors, the findings of this study should be interpreted with caution. Larger-scale studies are needed to validate and confirm the associations observed in this research. Lastly, we only assessed one activity (i.e., counter-movement jump), which may not necessarily be representative of other short-term, all-out activities such as sprints or changes of direction. Different physical tasks may require different physiological adaptations, and focusing solely on jump performance may limit the generalizability of the findings to other athletic endeavours.

## 7 Conclusion

This study demonstrates that lower limb muscle volume and length were associated with muscle power production during a counter-movement jump in adolescents aged between 16 and 19 years. Interestingly, these anthropometric factors were more relevant in males (where the correlations were positive) than in females (where the correlations were negative). However, the sex-differences in jump performance variables were still present after normalization by muscle volume and length, suggesting that other factors such sex-hormone levels (mainly testosterone) and muscle architecture and muscle fibre type distribution could also contribute to sex-differences in jumping performance.

The outcomes of this study offer guidelines for sports science coaches when planning muscle training activities. From a practical perspective, as lower limb length cannot be modified by training, lower limb muscle power training for adolescents should be focused on the development of muscle volume. The obtaining of higher muscle volumes with training will be more relevant for males than for females, as females with moderate values of thigh muscle volume may still exhibit high muscle performance jump height values.

## Data Availability

The original contributions presented in the study are included in the article/Supplementary Material, further inquiries can be directed to the corresponding authors.
